# How dynamic capabilities enable Chinese SMEs to survive and thrive during COVID-19: Exploring the mediating role of business model innovation

**DOI:** 10.1371/journal.pone.0304471

**Published:** 2024-05-31

**Authors:** Wenjun Huang, Takeyasu Ichikohji

**Affiliations:** Graduate School of Economics and Management, Tohoku University, Sendai City, Japan; University of Central Punjab, PAKISTAN

## Abstract

As a response to the damage caused by the spread of COVID-19, the Chinese government has implemented severe quarantine measures that have greatly affected the operational patterns of small and medium-sized enterprises (SMEs). This paper explores the critical role of dynamic capabilities (DCs) in helping Chinese SMEs manage crises, adjust their business strategies, and mitigate the uncertainty caused by the epidemic. Although the importance of DCs in promoting organizational resilience is well recognized, academic research on their specific contributions to business model innovation (BMI) and SME performance improvement during crises remains scarce. Our study fills this gap by pioneering the development and empirical testing of a microintegrated mediation model linking DCs, BMI and organizational performance. By surveying 257 Chinese SMEs severely affected by a pandemic, we verify our hypotheses using partial least squares structural equation modeling (PLS-SEM). Our results strongly show a positive relationship between DCs and BMI and SME performance. In addition, we found that BMI plays a partial mediating role in the interrelationship between DCs and SME performance. Our findings clarify the critical role of BMI as a channel through which SMEs’ DCs can be transformed into higher performance in the face of sudden crises. Thus, our results not only contribute to the broader discussion of strategic management and organizational theory but also provide theoretical and practical insights into the mechanisms by which SMEs can increase their flexibility and resilience in a crisis. Thus, our results not only contribute to the broader discussion of strategic management and organizational theory but also provide theoretical and practical insights into the mechanisms by which SMEs can increase their flexibility and resilience in a crisis. Importantly, this study suggests policy and market strategies that can support SMEs in leveraging DCs and BMI for sustained performance, thereby contributing valuable insights for policymakers and business leaders aiming to fortify economic stability and growth in the face of global health emergencies.

## Introduction

The uncertainty created by the COVID-19 pandemic and subsequent embargo policies has created unprecedented business challenges for Chinese small and medium-sized enterprises (SMEs), which have a weak resource base [1, 2]. Existing research suggests that SMEs can face and address external disruptions through business model innovation (BMI) [3]. This kind of BMI, which is based on changing the original value proposal of the firm, the way it is created and delivered, has the potential to help face disruptions and survive difficult times. After the pandemic, there has been a rapid increase in the literature on the intersection of BMI and SME survival and growth in the COVID-19 era. For example, [4], after studying the BMs of six SMEs in traditional industries during the COVID-19 pandemic, it was indicated that open innovation can help to promote BMI among SMEs in traditional industries during challenging times, such as during the pandemic [5]. They examined how BMI affects the strategic resources, key drivers, and processes of SMEs in the food and beverage industry from a causality perspective, thereby influencing performance to navigate through environments fraught with turbulence and uncertainty, such as COVID-19 [6]. Using five SMEs in Austria, Germany, and Liechtenstein demonstrated that temporary BMI to address the pandemic can allow SMEs to successfully overcome the crisis and improve performance.

In China, SMEs occupy an essential position and are the backbone of the national economy, contributing 50% of tax revenue, 60% of GDP (gross domestic product), 70% of technological innovation, 80% of jobs and 90% of the number of enterprises. Therefore, their failure during the COVID-19 pandemic will not only have a direct impact on the economic losses of their business partners but also have a direct or domino effect on the country and industries [7]. While SMEs are considered more timely and flexible in responding to sudden situations [8, 9], the intensity of the COVID-19 pandemic, along with unprecedented mobility restriction policies, has severely disrupted established business models (BMs), and novel studies are needed to better comprehend the utility of BMI in times of economic stagnation. However, Chinese SMEs are susceptible to outside interference because of their insufficient capital reserves, small size, poor access to credit, and low access to financial capital [10, 11]. Despite their apparent sensitivities, SMEs may be in a particularly dangerous position in times of sustained crises of unprecedented magnitude and embargo policies fraught with uncertainty, making it difficult for them to engage in BMIs when their original BMs become ineffective, which may require unique strategies, processes, and competencies to be explored. Therefore, further research is needed on what capabilities Chinese SMEs have deployed with respect to the COVID-19 pandemic to enable BMI to address the impact of this unprecedented disruption.

To this end, dynamic capabilities (DCs) provide guiding principles for exploring how BMI can be facilitated in unstable and unpredictable market conditions, such as those created by the COVID-19 pandemic, and what kinds of DCs can help enable BMI. We believe that DCs provide a mechanism through which SMEs can deploy the full range of resources available to them to adapt to disruptions. Specifically, DCs are advanced through the activities of sensing (identifying opportunities and threats), seizing (valuable opportunities), and transforming (resource and strategy reconfiguration). Existing studies indicate that these sensing, seizing, and transforming capabilities can help SMEs with BMI, namely, an organization’s capacity to continuously adapt its existing BMs to evolving business environments and customer needs to create long-term business value. Despite the recent interest of scholars in the topic of DCs, empirical studies on the correlation between DCs and BMI among SMEs do not exist within the setting of COVID-19. In the recent literature on COVID-19 and DCs, DCs have been studied in the context of social capital [12], social media analytics and competitive intelligence [13], the use of internal and external resources [14], digitization [15–18], and entrepreneurial ecosystems [19]. Therefore, more studies are required to determine how DCs in SMEs help organizations achieve BMI for performance against the background of disruption (COVID-19). In response to this study gap, we conducted a questionnaire survey of 257 Chinese SMEs. Using the partial least squares structural equation modeling (PLS-SEM) methodology, we analyzed the collected data to reveal the specific linkages between DCs, BMI and performance in SMEs.

Our study has four unique contributions. First, building on previous research on the relationship between DCs and BMIs outside crisis conditions, we provide the first empirical exploration of how DCs and BMIs interact within SMEs during the COVID-19 crisis. This study broadens the understanding of the interconnection between DCs and BMIs during turbulent times. Second, our study emphasizes the important role of DCs and BMIs in helping Chinese SMEs cope with the challenges and opportunities brought about by COVID-19. Unlike earlier studies that examined larger SMEs or start-ups in developed economies such as Germany, the UK, and the US, our study focuses on SMEs in China, a country with an expanding economic influence. We illustrate how the specific DCs of Chinese SMEs—in particular, their ability to sense, seize, and transform—play a key role in BMI, helping them cope with the downturns brought about by COVID-19 and utilize the unique opportunities that COVID-19 presented to create value. Third, by focusing on the actions of Chinese SMEs, we enrich the dialog at the intersection of SME research by illustrating how resource-constrained firms can not only withstand the direct impacts of the epidemic but also realize lasting transformation by exploiting these conditions. Fourth, we provide important insights from SMEs operating in China, which is particularly important given China’s stringent quarantine and movement restriction policies in response to COVID-19. These insights are crucial for understanding the recovery mechanisms of resource-limited SMEs in such crises.

In the following sections, we explore in depth the theoretical underpinnings of DCs and BMIs, presenting our research hypotheses and conceptual framework. Following the methodology and data analysis sections, we will discuss our findings, highlighting the strengths, weaknesses, and approaches for future research.

## Theoretical foundations

### Chinese SMEs during the COVID-19 pandemic

The COVID-19 pandemic has introduced unprecedented challenges to SMEs in China [20]. The measures taken by the government to control the spread of the virus, including city lockdowns, the enforcement of quarantines, halts in work, and restrictions on travel, created significant operational and financial challenges for SMEs [21]. These interventions, while necessary for public health, have resulted in discontinuity in supply chains, shortages in raw materials and a marked decline in consumer demand. Due to their relatively small scale and limited financial resources, SMEs face serious challenges [22, 23]. These constraints exacerbate their vulnerability to financial instability and potential bankruptcy, in contrast to the resilience demonstrated by large firms with strong infrastructure and economies of scale [2].

Despite these disadvantages, this does not mean that SMEs were helpless during the epidemic. Evidence suggests that disasters such as the COVID-19 pandemic also offer unique opportunities to SMEs characterized by agility and flexibility [24]. A McKinsey survey of 200 SMEs affected by COVID-19 in 2020 showed that more than half of business executives believe that pandemics present new business opportunities [25]. [26] also supports this view by pointing out that SMEs that effectively utilize the opportunities presented by pandemics can improve their performance and achieve sustainable growth. In addition, SMEs have flatter organizational structures that provide greater flexibility in developing and implementing strategies. Studies in the literature [27, 28] suggest that this flexibility allows SMEs to adapt more effectively and quickly than their rigid counterparts and take the initiative in change during a crisis. In conclusion, although SMEs face many challenges during the current crisis, their uniqueness and strategic flexibility provide them with opportunities to save the day. The key lies in their ability to identify and seize emerging opportunities (DCs), remain adaptive in their strategic planning, and innovate their BMs in response to the dynamics of the crisis.

However, despite theoretically recognizing the potential advantages of these dynamics, existing research on Chinese firms coping with the COVID-19 pandemic has focused on topics such as the role of social media and competitive intelligence in DCs [13], the effect of the pandemic on the fiscal system [29–32], and the impact of organizational resilience on SME performance [16], among others. There is still a large gap in understanding how DCs interact with BMI to influence the performance of SMEs in China during this unprecedented crisis. Our study seeks to address this gap by pioneering the linking of DCs with BMIs in such crises, revealing a critical yet underexplored aspect of strategic management in turbulent times.

### Business model innovation during the COVID-19 pandemic

A BM is the foundation of a company’s operations and value logic and determines its success or failure by answering key managerial questions about customer identification, value creation, and profit generation [33, 34]. A BM consists of three interdependent elements—value proposition, value creation, and value capture—which together form a cohesive strategy for delivering value to customers and ensuring profitability [6, 35]. Of these, the value proposal describes the company’s solution for satisfying client needs and is a foundation upon which the BM is built. Value creation describes how a firm uses its resources and capabilities to create value, focusing on the operational elements of products and services [36]. Value capture elucidates the revenue mechanism, illustrating how a firm translates its value proposal and creation efforts into financial success [37].

BMs are not static. Historically, the evolution of BMs has often been triggered by crises, competitive pressures, technological advances, or managerial reforms [33, 38, 39]. The COVID-19 pandemic was one such catalyst that made many traditional BMs obsolete and faltering in the new economic landscape. To survive and grow, companies must engage in BMI [40]. BMI is characterized by significant alterations in the fundamental components and structure of a company’s BM, facilitating the reimagining of how value is created and captured. [37, 41, 42], enabling firms to redefine the mechanisms of value creation and capture. Thus, coping with the complexities of pandemics can be a key strategy.

Although BMI is likely to play a key role during a pandemic, we identified a distinct gap in the literature: a lack of in-depth analysis of BMI practices in the COVID-19 era. This negligence suggests an oversight of contemporary insights into how firms, especially SMEs, reconfigure their operational logic and value proposals in response to crises. Prior research to this study has emphasized that BMI has the potential to provide firms with a competitive advantage and facilitate their adjustment in highly uncertain environments [6, 43]. However, the specific dynamics of BMI throughout the COVID-19 pandemic, along with its effects on the resilience and growth of SMEs, remain underexplored.

Numerous studies have begun to fill this gap. For example, [6] conducted a detailed longitudinal study of five SMEs in Austria, Germany and Liechtenstein. Similarly, [5] conducted an extensive survey of SMEs in the food and beverage sector, while [44] conducted a detailed cross-comparison of 10 culturally creative SMEs. Together, these studies emphasize that SMEs with the ability to innovatively adapt their BMs not only survive the crisis more effectively but are also better prepared for postpandemic growth. This evidence suggests that BMI is not only a survival tactic but also a strategic approach to fostering long-term resilience and competitive advantage.

However, implementing BMI in the context of the COVID-19 pandemic crisis is a challenging task, especially for SMEs suffering from resource constraints and limited access to financing [45, 46]. Moreover, the literature fails to provide guidance on facilitating BMI for Chinese SMEs during the crisis, especially regarding the antecedents of successful BMI. Effective BMI requires a profound comprehension of market trends, consumer requirements, and the evolution of technological advancements [47, 48]. The literature suggests that the DC framework is expected to support SMEs in addressing these BMI challenges by making them constantly aware of market changes and adapting to uncertain environments [49, 50]. DCs argue for changing BMI from a static viewpoint to one that is dynamic. This perspective argues that DCs enable SMEs to continuously modify their BMs in reaction to evolving market circumstances, customer demands, and crises [46, 49, 50]. However, the application of the DC framework to support BMI during the COVID-19 pandemic remains underexplored and represents a significant gap in the literature. By incorporating DCs into the BMI process, this study not only fills this important gap but also provides new insights into how SMEs can dynamically manage and allocate resources to enhance resilience and promote innovation during turbulent times.

### Dynamic capabilities during the COVID-19 pandemic

As discussed previously, to explore how BMI unfolded during the COVID-19 pandemic, scholars have increasingly drawn on insights from the field of DCs [51, 52]. Unlike ordinary capabilities that sustain an organization’s day-to-day operations and basic business through routine activities [53], DCs emphasize adapting to changing and risky business environments by strategically reconfiguring a firm’s existing resource base, processes, capabilities, and knowledge into new types of products/services and processes [35]. While these adaptive capabilities that are sensitive to external changes depend to a significant extent on the instability of the environment, because of their nature, they are not designed to facilitate continuous organizational change under stable market conditions. Therefore, extreme examples of force majeure, such as COVID-19, provide an excellent context for studying how SMEs can develop DCs to cope with the variations and threats posed by COVID-19 [54]. A recent study confirmed the positive link between DCs and firms’ competitive advantage and organizational resilience in unpredictable business contexts such as those affected by COVID-19 [55–58].

Drawing on [59] essential insights, DCs have been conceptualized as a firm’s ability to integrate, build, and reconfigure internal and external capabilities to respond to a rapidly changing environment. Throughout the pandemic, DCs have significantly contributed to BMI primarily through three interrelated capabilities: sensing, seizing, and transforming capabilities. Sensing capabilities focus on the strategic recognition and understanding of opportunities and threats that arise in a pandemic [35]. This capability is critical for SMEs because it involves identifying changing market demands, sensing the consequences of pandemics and potential blockades that may interrupt their production, operations and sales processes, and capturing cues from suppliers and competitors [60, 61]. This is the basis for SMEs to anticipate the necessary adjustments in strategic resource allocation, enabling them to remain agile in the face of environmental change. Seizing capabilities are not only about recognizing opportunities and threats during a pandemic but also about transforming perceived opportunities and challenges into valuable BMIs through effective decision making and resource allocation [35]. These capabilities are manifested in areas such as making strategic investments in new technologies and new ideas, which are critical to capturing identified opportunities [35]. According to [35], once an opportunity is identified, firms must not only innovate in product development but also allocate significant resources to these innovations to ensure a competitive advantage. Transforming capabilities refer to firms’ flexibility in adapting and restructuring their assets and organizational structures in response to perceived opportunities and market changes [35]. This involves coordinating organizational resources, adopting new processes, and developing new competencies so that firms can respond to market changes more flexibly and dynamically. Transforming capabilities are essential for maintaining the evolutionary adaptability of SMEs, enabling them to overcome inertia and barriers to innovation [62] and thus optimize and adjust their BMs to meet altering market settings. Essentially, through these capabilities, DCs determine the capacity of SMEs to recognize emerging opportunities and guide their strategic direction in a crisis scenario, making BMI the main outcome of DC utilization. These capabilities have become even more important, especially within the context of the COVID-19 pandemic.

## Hypothesis

### Business model innovation and performance

Previous research has indicated that BMI has emerged as a crucial strategy for companies to manage economic uncertainty during the COVID-19 pandemic [4, 5, 63]. BMI enables firms to efficiently reallocate existing resources to meet changing customer demands, optimize their cost structure, and explore new sources of revenues to sustain their operations during economic downturns. This innovative strategy helps firms create a new niche that is not covered by competitors [64]. According to [65], companies aiming to enhance their long-term performance are required to innovate their BMs. Through BMI, firms can not only identify customer needs more accurately, improve product quality and performance, and minimize production costs but also commercialize new technologies, ideas, and strategies through BMI to ensure competitive advantage [62].

The literature has theorized the paths through which innovation in the three components of the BM affects firm performance: value proposal, value creation, and value capture[35]. Innovations in value proposal enable firms to broaden the scope and mode of product and service offerings [66] to meet changing customer needs, which is critical for maintaining and improving performance in times of crisis. Conversely, innovations in value creation and value capture help firms maximize the effectiveness of their value proposal or explore new value proposals through novel activities and techniques. Such innovations not only help companies develop novel sources of revenue but also improve their cost structure, efficiency and profitability [67].

Despite these theoretical claims, empirical research examining the influence of BMI on SME performance, especially during the COVID-19 pandemic, has tended to adopt a qualitative approach [4, 5, 68, 69]. This preference for qualitative over quantitative approaches leaves a clear statistical gap. Moreover, existing studies have reached mixed conclusions on the relationship between BMI and performance in SMEs, with results ranging from positive to neutral and, in some cases, even undefined [70–72]. This inconsistency highlights the need for further empirical research, especially in environments such as China, where SMEs are vital to the economy and where the impact of external crises such as COVID-19 may be different from that of developed economies. To address these shortcomings, this research seeks to empirically test the effect of BMI on the performance of Chinese SMEs during the COVID-19 pandemic through the following hypotheses:

*H1a*: *Innovations in value proposals have direct and significant impacts on firm performance*.
*H1b*: *Innovations in value creation have a direct and significant impact on firm performance*.
*H1c*: *Innovations in value capture have a direct and significant impact on firm performance*.

### Sensing capabilities

Sensing capabilities denote the capacity of a company to identify opportunities and threats, both within the organization and in the external environment [73]. During the COVID-19 pandemic, even though the flat organizational structure and lower bureaucracy of Chinese SMEs grant them greater flexibility, making them more accessible to the market and customers [74], they still face challenges in understanding and responding to market demands, competition, and technological changes in a timely manner owing to their lack of resources and the low managerial acumen of their leaders [75]. However, sensing capabilities can compensate for this challenge by enhancing SMEs’ sensitivity to external environmental changes, mainly manifested in helping SMEs gain insight into subtle market changes, whether they have customer needs, technological trends, or competitive structures. Research by [76] indicates that this can stimulate value creation for enterprises. Therefore, sensing capabilities are vital for value creation. Studies by [35, 77] have shown that strong sensing capabilities in DCs can actually promote innovation in value capture. This is because companies with strong sensing capabilities can monitor changes in the business environment more flexibly than competitors can, adjust their activities in a timely manner, and evaluate the sustainability of current value capture [78]. Moreover, during the COVID-19 pandemic, Chinese SMEs faced fierce competition and an uncertain environment. A keen market sense can timely detect potential customer needs, new policy trends, etc., which can help them formulate appropriate strategies and actions, seize opportunities, create differentiation, and increase market share and revenue [79]. Based on this, we propose the following hypothesis:

*H2a*: *Sensing capabilities positively influence firm performance*.
*H2b*: *Sensing capabilities positively influence value creation innovation in BMI*.
*H2c*: *Sensing capabilities positively influence value capture innovation in BMI*.
*H2d*: *Value creation innovation and value capture innovation play a partially mediating role between sensing capabilities and firm performance*.

### Seizing capabilities

Seizing capabilities include the ability to deploy resources effectively to seize opportunities, meet needs and respond to challenges [73]. In a rapidly changing market environment (such as the COVID-19 pandemic), it is quite challenging for SMEs to achieve stable and sustainable profits. Studies have indicated that to profit in a volatile business landscape, managers should develop a series of strategies to gain short-term competitive advantages. This short-term advantage is achieved by seizing opportunities [80]. Seizing capabilities allow businesses to quickly select business opportunities that match their environmental context and advantages [35]. Therefore, seizing capabilities are often linked with good company performance. Considering the natural limitations of SMEs in terms of scale, funds, and technology [75], they might resist investing resources in new opportunities and ideas when responding to crises. However, through seizing capabilities, SMEs can enhance the efficiency and collaborative capacity of their resources, both internally and externally. These, in turn, enable them to integrate and guide their own or their partners’ resources, crafting new value propositions meticulously [35]. Additionally, seizing capabilities also help SMEs establish and maintain solid connections with external stakeholders, including suppliers, customers, competitors, and regulators. This paves the way for the inflow of critical information, increased support for value creation, and enhanced trust, thereby reducing transaction expenses and potential risks. Notably, although Chinese SMEs faced greater risks and resource constraints during the COVID-19 outbreak than before, their inclusive corporate atmosphere has created an environment where employees can shine and demonstrate their abilities and value [81]. Therefore, the seizing capability of SMEs contributes to value creation for the company [35]. Based on this, we propose the following hypothesis:

*H3a*: *Seizing capabilities positively influence firm performance*.
*H3b*: *Seizing capabilities positively influence value proposal innovation in BMI*.
*H3c*: *Seizing capabilities positively influence value creation innovation in BMI*.
*H3d*: *Value proposal innovation and value creation innovation play a partially mediating role between seizing capabilities and firm performance*.

### Transforming capabilities

According to [35], transforming capabilities refer to the ability of enterprises to keep their system elements and strategy consistent and coherent. It helps enterprises maintain long-term competitiveness by adapting to environmental changes. The capabilities associated with restructuring and transformation enable firms to generate Schumpeterian rents [82]. In addition, transforming capabilities are rooted in a firm’s complex organizational processes, which make them difficult for competitors to imitate and, therefore, likely to generate Ricardian rents [59]. Therefore, transforming capabilities help firms obtain superior performance. Moreover, the myopic, inert, and locked-in nature of SMEs [62] limits their ability to identify and acquire new ideas, potentially exerting a considerable adverse effect on the performance of the firm [83]. By reallocating various resources within the firm during the COVID-19 period, transforming capabilities enable the firm to overcome path dependence and corporate inertia [84] and thus empower the firm to develop new value proposals [85] and find new directions for the firm. For example, as computer capabilities evolved, IBM leveraged its decades of accumulation to transition its focus from being a hardware supplier to being a service provider. This strategic shift has led to significant success in consulting, IT maintenance, and various other services [86]. Based on this, we propose the following hypothesis:

*H4a*: *Transforming capabilities positively influence firm performance*.
*H4b*: *Transforming capabilities positively influence value proposal innovation in BMI*.
*H4c*: *Value proposal innovation plays a partially mediating role between transforming capabilities and firm performance*.

**Fig 1:**
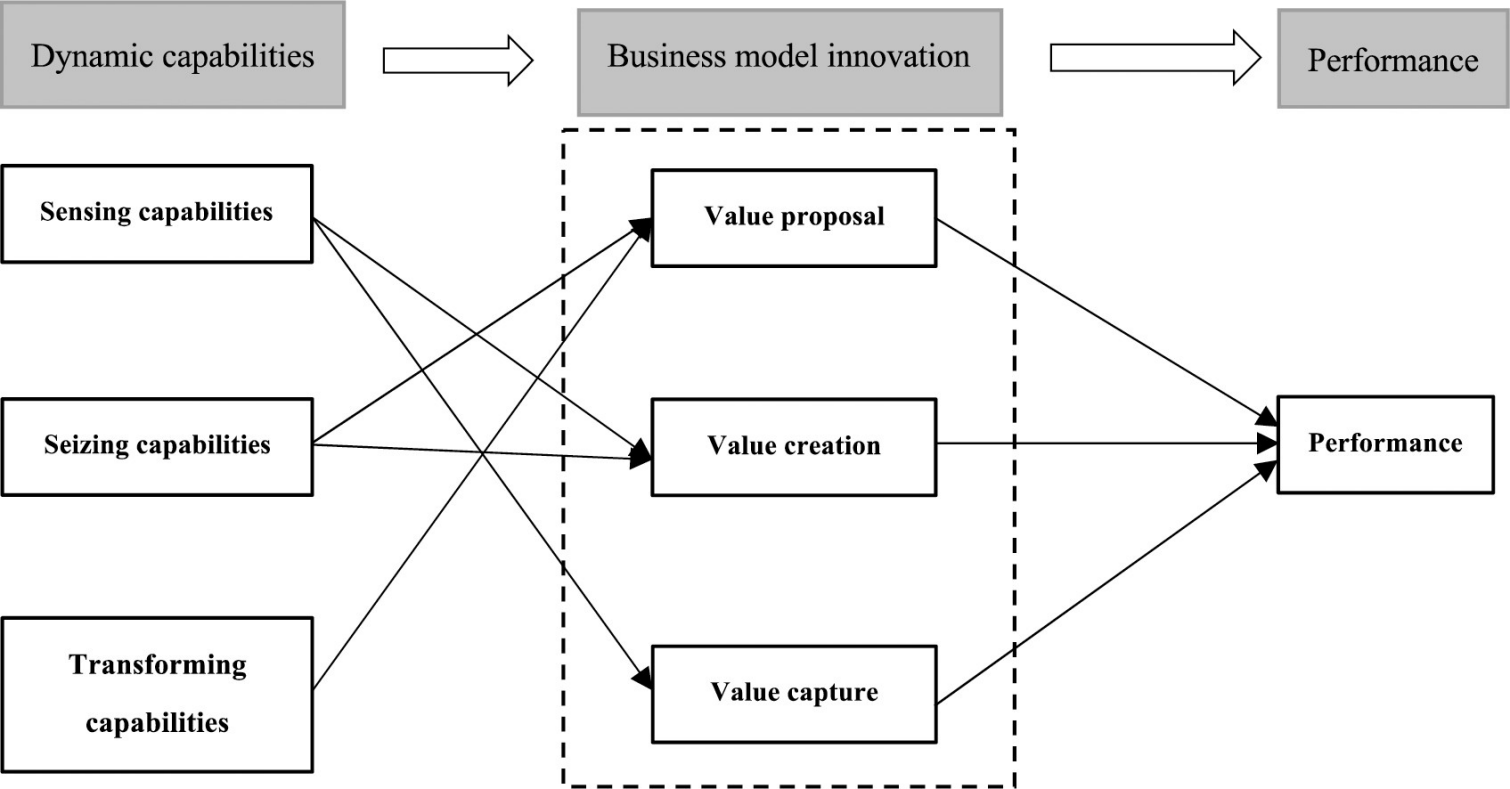
A multitheoretical framework of dynamic capabilities and performance.

Therefore, our study builds on previous research on DCs and BMI by examining the connection between DCs and firm performance and, on this basis, to explain the mediating role of BMI. Hence, this research proposes the following multitheoretical framework, as shown in Fig 1.

## Methodology

### Data collection

To test our hypotheses, we conducted a survey targeting Chinese SMEs between February and March 2023 using the online questionnaire platform Wenjuanxing. The reason for surveying Chinese SMEs during this period was that it was on the eve of the announced end of China’s anti-epidemic measures, which provided an opportunity to test those SMEs that had survived the three-year epidemic-ravaged environment. In addition, China’s COVID-19 embargo policy was extremely severe and had a serious negative impact on SMEs across the country. This makes China an ideal time and place to explore how SMEs can survive the crisis. Considering the effect of COVID-19 and the consistency of the related blocking policies, we used a random sampling method to screen for SMEs with fewer than 1,000 employees from China’s well-known enterprise credit inquiry tool, Aiqicha (https://www.aiqicha.com/), and downloaded their contact information. Since our target population was Chinese SMEs, the survey had to be in Chinese; for this reason, we used back-translation techniques to guarantee the precision of the questionnaire. Specifically, we first translated the English items into Chinese and then asked PhD students with strong Chinese and English skills to translate them back into English. In addition, to ensure simplicity and meaningless bias, we invited 10 people with different educational backgrounds (high school, bachelor’s degree, master’s degree) and contexts (managers and business researchers) to preview the questionnaire and revise it based on their feedback. Ultimately, we designed a valid and reasonable questionnaire. To increase the response rate of the questionnaire, we offered some monetary incentives to the respondents. Despite the involvement of human participants, our study adhered to institutional and national ethical standards and therefore did not require explicit ethical approval. The study was limited to the distribution of questionnaires and did not involve human clinical trials or animal experiments; therefore, it complied with ethical guidelines, including the Declaration of Helsinki. All participants were guaranteed confidentiality, anonymity and voluntary participation and provided written informed consent. Out of 1000 distributed questionnaires, 310 were returned. After removing invalid responses, we obtained 257 valid questionnaires, which represents a valid recovery rate of 25.7%.

### Variables

To guarantee the measurement model’s validity, all our variable measurement scales are derived from previous confirmatory studies. If necessary, we will make some moderate adjustments according to the actual situation of Chinese SMEs. Where needed, moderate modifications were made to better align with the specific context of Chinese SMEs. Furthermore, this study mainly employs a 5-point Likert scale to assess each factor, with the scale defined as follows: “1 = I totally disagree,” “2 = I disagree,” “3 = I cannot tell,” “4 = I somewhat agree,” and “5 = I strongly agree.”

### SME performance

SME performance involves evaluating the success of an entire company from an overall organizational level rather than a single product or production line. Based on previous studies on SME performance measurement, we followed the practices of [87, 88] and measured Chinese SME performance in terms of return on assets, return on sales, total sales growth, and new product creation.

### Dynamic capabilities

In previous studies, we mentioned that DCs include three capabilities: sensing capabilities, seizing capabilities, and transforming capabilities. To measure these three subcapabilities, we adopted the measurement scale developed by [89]. Although this scale is not perfect, it has been used by multiple quantitative studies and has achieved good results.

### Business model innovation

We used items developed by researchers such as [90] and [37] to measure the three components of BMI: value proposal, value creation, and value capture.

### Control variables

To compensate for other variables that may affect DCs, BMI, and performance, we controlled for six variables that may affect company performance. They are the respondent’s age, education (EdU), experience (Exp), gender (Gen), position (Pos), and firm size (FS). Table 1 shows the descriptions of potential multi-item variables and measurement items.

**Table 1:** Quantitative measurement instrument.

Item	Question	Reference
Sensing Capabilities(SE) in DCs		[89]
SE1	We are well-versed with the market’s best practices.	
SE2	We stay informed about the latest market trends.	
SE3	We actively gather data on market conditions.	
SE4	We effectively source new market information.	
SE5	We monitor the activities of our competitors closely.	
SE6	We swiftly identify market shifts.	
Seizing Capabilities(SZ) in DCs		
SZ1	We adeptly assimilate external knowledge.	
SZ2	We discern which new information is beneficial for us.	
SZ3	We adeptly convert new tech insights into innovations.	
SE4	Present-day data catalyzes the birth of novel products/services.	
Transforming Capabilities(TR) in DCs		
TR1	By setting distinct roles, we drive effective change.	
TR2	Despite setbacks, our change initiatives remain persistent.	
TR3	Change decisions are unwaveringly followed.	
TR4	Historically, we’ve showcased our change management prowess.	
TR5	We manage daily tasks and change projects concurrently.	
TR6	We adjust change plans according to current needs.	
Value proposal(VP) in BMI		[37, 90]
VP1	The target audience has evolved.	
VP2	Our product/service range has been revamped.	
VP3	Our market stance has transformed.	
Value creation(VC) in BMI		
VC1	Core competencies have evolved.	
VC2	Key resources have been modified.	
VC3	Business procedures have undergone change.	
VC4	Our resource allocation strategy has been updated.	
VC5	The contribution of partners to value generation has been altered.	
VC6	Our distribution methods have been updated.	
Value capture(VCP) in BMI		
VCP1	Revenue strategies have undergone changes.	
VCP2	Costing methods have been adjusted.	
VCP3	Fixed expenditures have been modified.	
VCP4	Variable expenditures have seen revisions.	
Performance(P)		[87, 88]
P1	In the last three years, our return on assets surpassed industry norms.	
P2	In the past three years, our return outshone industry averages.	
P3	In the past three years, our sales growth exceeded industry standards.	
P4	In the past three years, our new product/service launch rate exceeded industry averages.	

### Data analysis procedures

This study uses a covariance-based structural equation technique, the PLS-SEM method, to validate the proposed hypothetical model. Unlike covariance-based SEM methods such as AMOS or LISREL, PLS-SEM does not enforce the data to follow a normal distribution and is good at handling and checking more complex models [91]. Therefore, it is particularly suitable for our study. In this study, we used a software specialized for SEM analysis, namely, SmartPLS, for data analysis, specifically version 4.0.8.7. According to the recommendations of [92], PLS-SEM analysis is divided into the following six steps: 1) structural model specification (defining the objectives of the study, describing the latent variables and their indicators, and clarifying directional relationships between the latent variables, which have been described in detail in the previous section), 2) data collection and processing, 3) measurement model assessment (assessing the reliability and validity of the constructs), 4) structural model assessment (assessing the relationships between the variables), 5) mediation effect assessment, 6) assessment of the overall fit of the model, and 7) interpretation of the results and conclusions.

## Results

### Descriptive statistics

Table 2 shows that most of the respondents were middle or senior managers (66.54%) or firm owners (24.9%). In terms of company size, companies with fewer than 50 employees are the most common (43.19%), followed by companies with 51–200 employees (30.74%). The respondents’ years of experience were mostly between 6 and 10 years (47.86%). In terms of data collection and sample characteristics, our questionnaire has high validity and representativeness.

**Table 2:** Sample distribution.

Gender	Frequency	Percentage	Age	Frequency	Percentage
Man	137	53.31%	21–30	72	28.02%
Female	120	46.69%	31–40	85	33.07%
**Education**			41–50	59	23.35%
Primary school	4	1.56%	51–60	38	14.4%
Middle school	11	4.28%	>60	3	1.17%
High school	26	10.12%	**Experiences**		
Specialized school	51	19.84%	1–5	61	23.74%
Undergraduate	108	42.02%	6–10	123	47.86%
Master	42	16.34%	11–15	53	20.62%
PhD	15	5.84%	16–20	12	4.67%
**Firm size**			>20	8	3.11%
<50	111	43.19%	**Position**		
51–200	79	30.74%	Company executives	22	8.56%
201–500	41	15.95%	Firm’s owner	64	24.9%
501–1000	26	10.12%	Mid-senior leadership	171	66.54%

### Multicollinearity tests

Since this study used cross-sectional data from a single source, there may be common methodological bias (CMB). To avoid affecting the results of our tests and to ensure the robustness of our analysis, we examined CMB through the following 3 dimensions. The first was Harman’s test of a single factor. The results demonstrated that a single factor explained merely 25.065% of the total variance, significantly beneath the 50% threshold anticipated by [93]. The second test was the variance inflation factor (VIF). All the VIFs were less than 2.179 (see Table 4 for details). This was less than what [91] predicted 3. The third was the correlation matrix method. [94] states that CMB exists if the correlation between major indicators exceeds 0.90. Nonetheless, as shown in Table 3, no correlation between any two indicators exceeded 0.90. These three methods demonstrated that our data did not contain severe CMB issues.

**Table 3:** Means, standard deviations and correlations among study variables.

Variables	Mean	s.d	1	2	3	4	5	6	7	8	9	10	11	12	13
**1 P**	4.006	0.860	1.000												
**2 SE**	3.757	0.863	0.472**	1.000											
**3 SZ**	3.670	0.966	0.477**	0.382**	1.000										
**4TR**	3.940	0.816	0.503**	0.365**	0.276**	1.000									
**5 VP**	3.678	1.014	0.411**	0.187**	0.260**	.0260**	1.000								
**6 VC**	3.661	0.898	0.440**	0.337**	0.298**	0.220**	0.300**	1							
**7 VCP**	3.844	0.867	0.442**	0.349**	0.324**	0.234**	0.302**	0.338**	1.000						
**8 Gen**	1.470	0.500	-0.015	0.017	-0.038	-0.016	0.035	-0.029	-0.109	1.000					
**9 Age**	3.280	1.060	-0.078	-0.141*	-0.124*	-0.152*	-0.037	-0.04	-0.042	0.146*	1.000				
**10 EdU**	4.690	1.242	0.043	-0.015	-0.039	-0.024	0.068	-0.025	0.005	-0.029	-0.047	1.000			
**11 Pos**	2.580	0.645	0.175**	0.175**	0.157*	0.086	0.202**	0.113	0.213**	-0.019	-0.127*	0.133*	1.000		
**12 Exp**	2.160	0.943	-0.091	0.027	-0.016	0.021	-0.079	0.009	0.006	-0.014	0.406**	-0.068	-0.04	1.000	
**13 FS**	1.950	1.037	-0.114	-0.036	-0.12	-0.012	-0.025	-0.03	-0.103	0.132*	0.174**	-0.065	-0.297**	0.164**	1.000

** Significant correlation at the 0.01 level (two-tailed).

* Significant correlation at the 0.05 level (two-tailed).

### Measurement model evaluation

We conducted calculations on the measurement model in SmartPLS 4.0.8.7, a software program with default settings. We thoroughly evaluated the measurement model in terms of reliability, convergent validity and discriminant validity. Reliability was assessed by measuring internal consistency, which was generally confirmed by Cronbach’s alpha, Dijkstra-Henseler’s rho (rho_A), composite reliability (CR) and average variance extracted (AVE). As shown in Table 4, all the constructs have Cronbach’s alpha, Dijkstra-Henseler’s rho (rho_A), CR values greater than 0.7 and AVE values greater than 0.5 [95, 96]. Therefore, the constructs in this study are sufficiently reliable. Convergent validity assesses the ability of a model to explain variation in variables. To determine convergent validity, we examined the factor loadings of the items of each construct. According to prior research, factor loading values should exceed 0.7 [92, 97]. Table 4 shows that the values of factor loadings for each item are greater than 0.7; therefore, convergent validity is confirmed.

**Table 4:** Measurement model scale results.

Constructs	Items	Factor loading	VIF
Sensing Capabilities(SE) in DCs (Cronbach’s alpha = 0.887, rho_A = 0.889, CR = 0.914, AVE = 0.639)	SE1	0.783	1.927
	SE3	0.792	1.966
	SE5	0.792	1.957
	SE6	0.804	2.076
	SE4	0.810	2.043
	SE2	0.815	2.082
Seizing Capabilities(SZ) in DCs (Cronbach’s alpha = 0.863, rho_A = 0.866, CR = 0.907, AVE = 0.708)	SZ3	0.830	1.976
	SZ2	0.842	2.009
	SZ4	0.843	2.093
	SZ1	0.851	2.000
Transforming Capabilities(TR) in DCs (Cronbach’s alpha = 0.866, rho_A = 0.870, CR = 0.899, AVE = 0.599)	TR6	0.743	1.674
	TR4	0.754	1.743
	TR1	0.773	1.820
	TR5	0.786	1.764
	TR3	0.791	1.849
	TR2	0.794	1.916
Value proposal(VP) in BMI (Cronbach’s alpha = 0.817, rho_A = 0.819, CR = 0.891, AVE = 0.731)	VP2	0.845	1.662
	VP1	0.856	1.936
	VP3	0.864	1.907
Value creation(VC) in BMI (Cronbach’s alpha = 0.888, rho_A = 0.900, CR = 0.914, AVE = 0.639)	VC3	0.770	1.907
	VC2	0.792	2.027
	VC4	0.796	1.979
	VC5	0.803	2.092
	VC1	0.804	1.886
	VC6	0.832	2.156
Value capture(VCP) in BMI (Cronbach’s alpha = 0.826, rho_A = 0.830, CR = 0.884, AVE = 0.657)	VCP4	0.795	1.687
	VCP3	0.799	1.715
	VCP2	0.807	1.733
	VCP1	0.840	1.826
Performance(P) (Cronbach’s alpha = 0.849, rho_A = 0.850, CR = 0.898, AVE = 0.689)	P3	0.794	1.671
	P2	0.822	1.893
	P1	0.843	2.049
	P4	0.860	2.179

Discriminant validity focuses on assessing the discriminability of the model. To evaluate this, we applied the Fornell–Larcker criterion and the heterotrait–monotrait (HTMT) ratio [91]. According to the established standards, the square root of the AVE should surpass the correlation values with other constructs, and the HTMT ratio should not exceed 0.9 [91]. Tables 5 and 6 show the results of the Fornell–Larcker method and the HTMT ratio. These results indicate that the measurement model’s unique validity is unproblematic.

**Table 5:** Discriminant validity analysis (Fornell–Larcker criterion, 1981).

	1	2	3	4	5	6	7	8	9	10	11	12	13
**1 P**	0.830												
**2 SE**	0.473	0.799											
**3 SZ**	0.480	0.386	0.841										
**4 TR**	0.504	0.369	0.276	0.774									
**5 VC**	0.451	0.353	0.310	0.223	0.799								
**6 VCP**	0.445	0.352	0.322	0.239	0.341	0.810							
**7 VP**	0.410	0.189	0.262	0.261	0.296	0.303	0.855						
**8 Age**	-0.077	-0.141	-0.126	-0.151	-0.041	-0.044	-0.037	1.000					
**9 EdU**	0.042	-0.015	-0.039	-0.026	-0.025	0.004	0.068	-0.047	1.000				
**10 Exp**	-0.089	0.027	-0.015	0.025	0.012	0.006	-0.079	0.406	-0.068	1.000			
**11 Gen**	-0.015	0.018	-0.038	-0.015	-0.033	-0.111	0.036	0.146	-0.029	-0.014	1.000		
**12 Pos**	0.177	0.172	0.158	0.085	0.113	0.210	0.202	-0.127	0.133	-0.040	-0.019	1.000	
**13 FS**	-0.113	-0.035	-0.122	-0.011	-0.033	-0.102	-0.025	0.174	-0.065	0.164	0.132	-0.297	1.000

**Table 6:** Heterotrait–Monotrait analysis.

	1	2	3	4	5	6	7	8	9	10	11	12
**1 P**												
**2 SE**	0.545											
**3 SZ**	0.559	0.438										
**4 TR**	0.584	0.419	0.319									
**5 VC**	0.509	0.382	0.342	0.253								
**6 VCP**	0.530	0.407	0.384	0.278	0.395							
**7 VP**	0.492	0.218	0.309	0.308	0.352	0.369						
**8 Age**	0.084	0.150	0.134	0.164	0.049	0.076	0.041					
**9 EdU**	0.046	0.031	0.068	0.037	0.076	0.005	0.075	0.047				
**10 Exp**	0.097	0.054	0.036	0.079	0.044	0.042	0.088	0.406	0.068			
**11 Gen**	0.047	0.053	0.046	0.027	0.041	0.121	0.039	0.146	0.029	0.014		
**12 Pos**	0.191	0.185	0.169	0.093	0.120	0.233	0.224	0.127	0.133	0.040	0.019	
**13 FS**	0.123	0.045	0.130	0.037	0.047	0.113	0.031	0.174	0.065	0.164	0.132	0.297

### Structural model evaluation

**Fig 2:**
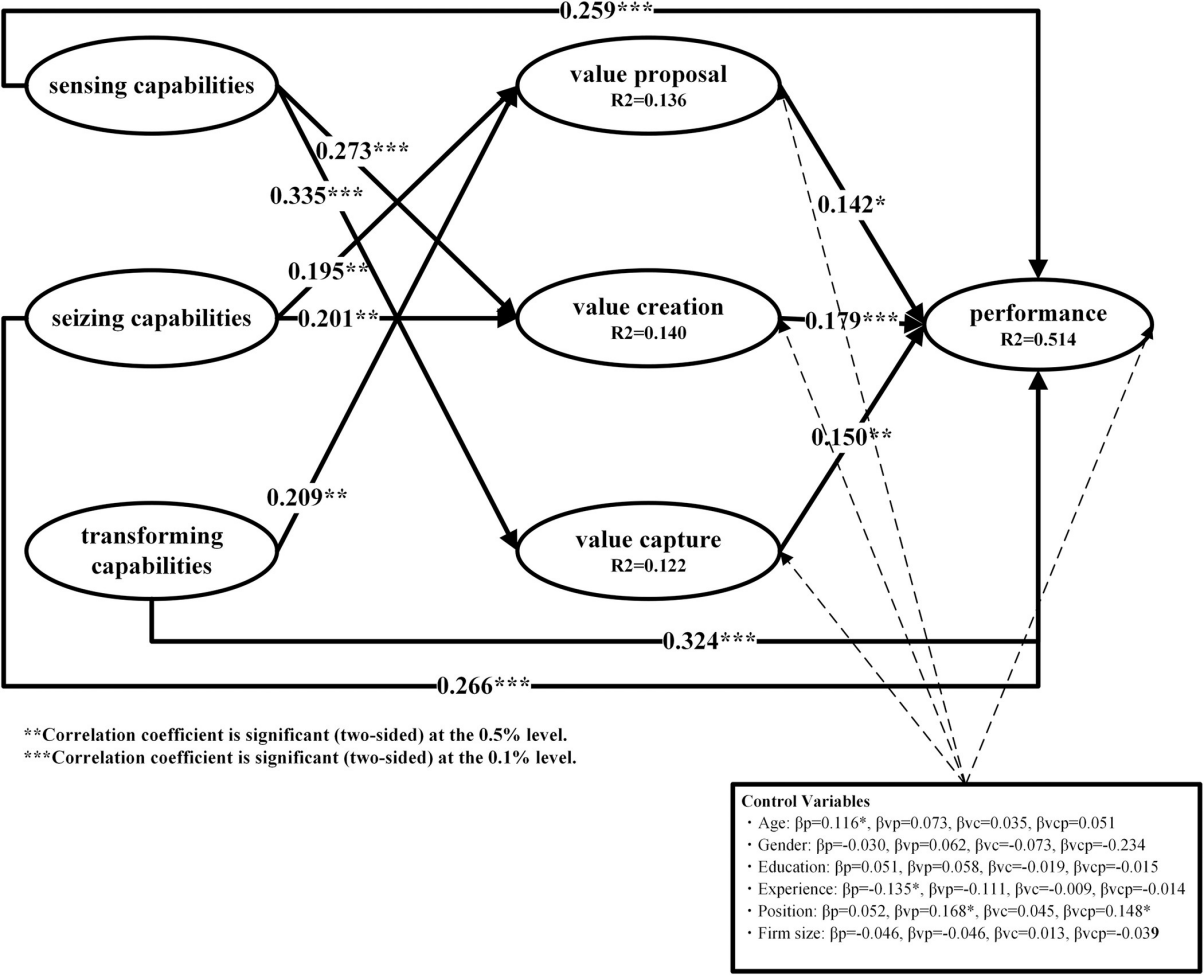
Result model.

After evaluating the measurement model, to assess the structural model and test the hypotheses, the bootstrap technique was used as described in the literature [98], employing 5000 random resamplings. Given that all the constructs in this study are reflective, the analysis focused on the endogenous coefficient (R^2^), the standardized path coefficient (β) and its statistical significance (T value), as well as the P value and confidence interval [91]. The findings from the PLS analysis of the structural model are depicted in Fig 2 and detailed in Table 7. The R^2^ value indicates the degree to which the model accounts for the variance in each variable. The R^2^ values in Fig 2 show that the structural model explained 51.4%, 13.6%, 14% and 12.2% of the variance in SME performance, value proposal, value creation and value capture, respectively. Since these values exceed the 10% threshold proposed in the literature [99], the model in this study has strong explanatory power. In addition to R^2^, the predictive validity of the model was further assessed by examining the Q^2^ value for the exogenous variables [91]. A Q^2^ value greater than 0 indicates predictive relevance, a value greater than 0.35 indicates high predictive validity, while a value less than 0 indicates a lack of predictive relevance [91]. The results of our analysis indicate that SME performance (Q^2^ = 51.4%), value proposal (Q^2^ = 13.6%), value creation (Q^2^ = 14%), and value capture (12.2%) have satisfactory predictive relevance.

**Table 7:** Direct effects analysis.

Relationships	β	T value	Bias corrected 95% confidence interval	Decision
**VP—> P**	0.142**	2.886	[0.049–0.242]	H1a supported
**VC—> P**	0.179***	3.339	[0.065–0.278]	H1b Supported
**VCP—> P**	0.150**	2.708	[0.038–0.256]	H1c Supported
**SE—> VC**	0.273***	3.697	[0.126–0.418]	H2b Supported
**SE—> VCP**	0.335***	5.048	[0.204–0.465]	H2c Supported
**SZ—> VP**	0.195**	2.891	[0.057–0.326]	H3b Supported
**SZ—> VC**	0.201**	2.876	[0.062–0.334]	H3c Supported
**TR—> VP**	0.209**	2.968	[0.077–0.350]	H4b Supported
**SE—>P**	0.259***	4.970	[0.152–0.357]	H2a Supported
**SZ—>P**	0.266***	5.017	[0.156–0.363]	H3a Supported
**TR->P**	0.324***	6.031	[0.224–0.438]	H4a Supported

Notes: n = 5000 subsamples.

**Correlation coefficient is significant (two-sided) at the 5% level.

***: The correlation coefficient is significant (two-sided) at the 0.1% level.

The analysis of path coefficients revealed significant positive impacts of value proposal, value creation, and value capture on performance, supporting hypotheses H1a, H1b, and H1c with the following path coefficients and statistics: β for value proposal = 0.142 with T = 2.886 and p < 0.05; β for value creation = 0.179 with T = 3.339 and p < 0.05; and β for value capture = 0.15 with T = 2.708 and p < 0.05. Furthermore, sensing capabilities significantly influenced value creation (β = 0.273, T = 3.697, p < 0.001) and value capture (β = 0.335, T = 5.048, p < 0.001), while seizing capabilities notably affected value proposal (β = 0.195, T = 2.891, p < 0.05) and value creation (β = 0.201, T = 2.876, p < 0.05). Additionally, transforming capabilities demonstrated a significant positive impact on value proposition (β = 0.209, T = 2.968, p < 0.05), thereby supporting hypotheses H2b, H2c, H3b, H3c, and H4b. Moreover, the influence of sensing, seizing, and transforming capabilities on performance was significant and positive (β for sensing = 0.259, T = 4.970, p < 0.001; β for seizing = 0.266, T = 5.017, p < 0.001; and β for transforming = 0.324, T = 6.031, p < 0.001), affirming hypotheses H2a, H3a, and H4a.

### Mediating effects test

To investigate mediating effects, we used the method recommended by [96], testing by comparing the direct and indirect effects between sensing capabilities, seizing capabilities, transforming capabilities, and performance. According to the results displayed in Tables 7 and 8, the indirect or mediated paths are significant (SE→VC→P; SE→VCP→P; SZ→VP→P; SZ→VC→P; TR→VP→P), and the direct paths are also significant (SE→P, SZ→P, TR→P). Therefore, the direct paths of sensing competencies, seizing capabilities, and transforming capabilities to performance are significant, while the indirect paths are significant, and the confidence intervals do not include zero; therefore, it can be concluded that the three dimensions of BMI partially mediate the relationships between the subcompetencies of DCs and SME performance.

**Table 8:** Mediation analysis (indirect effects analysis).

Relationships	β	T value	Bias corrected 95% confidence interval	Mediation type	Decision
**SE—>VC—> P**	0.049**	2.429	[0.014–0.090]	Partial	H2d Supported
**SE—>VCP—> P**	0.050**	2.340	[0.012–0.096]	Partial	H2d Supported
**SZ—>VP-> P**	0.028**	2.177	[0.006–0.056]	Partial	H3d Supported
**SZ ->VC—> P**	0.036**	2.078	[0.007–0.073]	Partial	H3d Supported
**TR—> VP—>P**	0.030**	1.963	[0.007–0.066]	Partial	H4c Supported

Notes: n = 5000 subsamples.

**Correlation coefficient is significant (two-sided) at the 5% level.

In addition, we further assessed the partial mediating effects of value proposal, value creation and value capture using the effect size (f^2^) index of the predictor variables suggested by [100]. [100] showed that f^2^ values of 0.02, 0.15 and 0.35 correspond to small, medium and large effect sizes, respectively. In addition to value proposal (f^2^ = 0.035), value creation (f^2^ = 0.053), value capture (f^2^ = 0.036), sensing capabilities (f^2^ = 0.038), seizing capabilities (f^2^ = 0.067), and transforming capabilities (f^2^ = 0.149) were also moderately predictive of firm performance. This finding suggests that value proposal, value creation and value capture indeed act as mediators to a certain degree in the dynamic between DC and SME performance.

### Model fit

Table 8 shows several fit indices used to assess the overall model fit. They are SRMR, d_ULS, d_G, chi-square (X^2^), and the relative fit index NFI. where SRMR measures the difference between the observed covariance matrix and the predicted covariance matrix. SRMR values lower than 0.08 indicate a good model fit [101]. d_ULS and d_G assess model fit by comparing the differences in covariance matrices. In general, lower values indicate a better fit. However, it is worth noting that their acceptable critical values need to be analyzed on a case-by-case basis. The chi-square test (X^2^) is a statistical test that is derived by comparing the difference between the observed covariance matrix and the model-inferred covariance matrix. Despite its importance, the chi-square test (X^2^) is very sensitive to sample size and is likely to produce significant results even if the sample size difference is small. Hence, it is advisable to complement the chi-square test (X^2^) with other fit indices. Finally, the relative fit index, NFI, is obtained by comparing the fit of the estimated model to that of the null model. A value closer to 1 indicates a better fit. The analysis in Table 9 shows that our model fits the data well [102].

**Table 9:** Goodness-of-fit tests.

	Saturated Model	Estimated Model
**SRMR**	0.048	0.066
**d_ULS**	1.760	3.389
**d_G**	0.658	0.700
**Chi-square**	952.151	1,002.038
**NFI**	0.804	0.794

Finally, in the last stage of the analysis, we added six control variables to the structural model for SME performance, value proposal, value creation, and value capture to test the robustness of the analysis. Fig 2 shows that these control variables have no substantial impact on the above variables, thus confirming the stability and reliability of the results.

## Conclusion and discussion

The objective of this study is to explore the insufficient research on the application of the DC framework in Chinese SMEs during the COVID-19 pandemic in promoting SMEs’ BMI to improve firm performance and survive the crisis. By conducting an empirical survey of 257 SMEs, we discovered a positive correlation between DCs (sensing, seizing, and transforming) and the three facets of BMI (value proposal, value creation, and value capture innovation). This finding highlights the critical need for swift and effective responses to unexpected environmental shifts and the implementation of strategies to cope with pandemics, meet customer demands, and secure value during turbulent times. In addition, we further investigated the relationships between these three dimensions of DCs (sensing, seizing, and transforming), BMI, and firm performance. The findings indicate that sensing capabilities, seizing capabilities, transforming capabilities, value proposal, value creation, and value capture are positively related to firm performance. This finding highlights the importance of developing firm capabilities, especially the significance of being alert to policy changes and resilient to crises, and suggests that SMEs are not helpless in a volatile environment and can rely on DCs and BMIs to survive exogenous crises. Finally, our mediation test results suggest that BMI is an important mediating mechanism (partial mediation effect) and that DCs (sensing, seizing, and transforming) can contribute to SMEs’ superior performance through the three dimensions of BMI. We argue that including this procedure further broadens our understanding of the function of BMI as an essential strategic channel, thereby demonstrating how DCs can influence SME performance rather than just exploring their direct effects.

### Theoretical contributions

There are three significant theoretical innovations in this study. First, our study enriches the DC literature by revealing the complex and dynamic interplay between DCs, BMI, and SME performance during the COVID-19 pandemic. While existing studies have elucidated the resilience offered by DCs during this period [103, 104], it is not clear whether DCs improve SMEs’ performance during the pandemic period through BMI. Through a comprehensive survey of 257 Chinese SMEs, our study elucidates the critical role of DCs in crisis management and BMI. This is consistent with findings in the literature [105, 106] that DCs play a key role in fostering innovation and enhancing SME performance. In contrast to studies by [38, 52, 68], our study focuses more on China’s unique policy environment and its impact on firms’ DCs and BMI strategies, revealing how China’s rigorous epidemic prevention measures provide firms with an unprecedented opportunity to readapt their BMs, which is less commonly seen in Western environments. This insight emphasizes the importance of cultural and market differences when deploying DCs, demonstrating the distinctive strategic adaptation and resilience mechanisms of Chinese SMEs during the crisis.

Second, our study provides novel perspectives on BMI drivers for SMEs in pandemic environments. Based on the theory of DCs and through empirical analyses, we identify and define ’sensing capabilities’, ’seizing capabilities’ and ’transforming capabilities’ as the main drivers of BMI. Integrating these capabilities into the literature on BMI in times of crisis has helped to shape the understanding of BMI. Our results are in line with the literature [107], suggesting that innovating BMs through DCs in response to global public crises is effective and necessary. Contrary to studies that view BMI as a systematic and structured concept [68, 89], our study views BMI as a series of innovations in different BM dimensions and focuses on which factors in DCs (sensing, seizing, transforming) can contribute to innovations in these BM dimensions (value proposal, value creation, and value capture). On this basis, we identified that different BM dimensions require different DCs. Unlike studies that consider DCs and BMI as a total concept [108, 109], our study not only expands the meaning and scope of DC theory [35] but also offers a more thorough viewpoint on identifying the drivers and paths of BMI [110].

Third, our study extends the main understanding of the connection between DCs and SME performance during crises by revealing the mediating role of BMI dimensions between DC subcapabilities and SME performance [75]. Our results generally affirm the positive influence of DCs on SME performance [74]. However, they also highlight BMI as a crucial mediator in this relationship, a factor that cannot be ignored. This insight suggests that merely cultivating and utilizing DCs without their strategic application through BMI may not fully realize the potential performance improvement of SMEs. Furthermore, by integrating DCs and BMI in the context of SME crisis management, our research also enriches the SME innovation discourse by addressing, to some extent, the challenge of difficult innovation that has troubled SMEs for many years [111]. In conclusion, in an increasingly globalized and uncertain world, our research offers valuable opinions and guidance for SMEs in China and globally.

### Managerial implications

Our survey illustrates several practical strategies for practitioners aiming to more effectively withstand exogenous crises. First, in situations of increased uncertainty in general, such as that experienced during the COVID-19 pandemic, we advocate that entrepreneurs and managers engage in efforts to cultivate DCs to enhance organizational awareness and sensitivity to environmental change. This strategic focus not only enhances firms’ adaptability and reactivity to external changes but also emphasizes the need for careful risk assessment of COVID-19. It also emphasizes the need for a sophisticated risk assessment of COVID-19, i.e., the need for companies to assess and seize emerging opportunities wisely, balancing the expected benefits with the associated risks. In addition, fostering DCs helps to increase organizational flexibility by enabling firms to move away from stereotypes and accept innovative practices and traditions.

Second, our study revealed that SMEs still face serious constraints in gathering market information. To alleviate this problem, SME managers must utilize their intrinsic strengths and explore opportunities beyond the traditional model. By employing DCs—i.e., sensing, seizing, and transforming capabilities—businesses can quickly identify and apply underemployed resources to realize new BMs. By employing DCs—i.e., sensing, seizing and transforming capabilities—businesses can quickly identify and apply underutilized resources to enable new BMs. This approach is particularly effective for SMEs and start-ups, especially in the logistics and technology sectors, which can withstand market volatility through digital integration and BMI not only through DCs but also beyond them. In addition, we found that implementing BMI during a crisis goes beyond a mere survival strategy. Not only does it help companies survive, but it also increases social benefits, improves organizational reputation, and paves the way for sustainable growth. In the case of emergencies, rapid and responsible innovation can help safeguard public health and save lives [112, 113]. Therefore, we recommend that business managers of all types maintain a sense of innovation and action in moments of crisis to carry out valuable and meaningful BMIs.

Third, to successfully implement BMI in the context of turbulence, managers must recognize that the various dimensions of BMI are affected to different extents by different DCs. Capabilities that facilitate value creation innovation are not necessarily aligned with those that favor value capture innovation. Matching specific capabilities with targeted BMI dimensions can significantly improve organizational performance and innovation success. In addition, we recommend that firms operating in Chinese jurisdictions remain sensitive to changes in government policies during and after the crisis. Business operations not only depend on the policy framework but are also vulnerable to its dynamic nature. Timely and accurate interpretations of policy changes and their integration into business strategies are crucial for improving operational efficiency or reducing costs. Finally, we believe that firms with limited resources should take an optimistic approach to the crisis. Viewing periods of crisis as windows of opportunity to cultivate can pave the way not only for survival but also for prosperity.

### Limitations

This study has numerous limitations and sets the stage for future investigations. First, this study was conducted on the eve of the end of the crisis, and by focusing on a single period, this study may not have been able to fully represent the changes triggered by the COVID-19 pandemic. To remedy this shortcoming, future research needs to follow the same SMEs continuously to obtain in-depth knowledge of the similarities and differences in the evolution of SMEs’ BMs before, during, and after the COVID-19 pandemic, as well as other factors that may have affected BMs. Furthermore, the scope of this study was restricted to SMEs operating within China. Although our theories, hypotheses, and measurement tools are generalizable, our findings may not fully and accurately capture the effect of DCs and BMIs on SMEs’ performance during a crisis period due to the influence of cultural or geographical differences. Therefore, future studies could conduct large-scale tests on different firms in different countries to test the accuracy of our model.

Second, this study only considered the mediating role of BMI, which may have neglected broader environmental factors that have a significant impact on firms’ operations. Future research could enrich our understanding by examining the mediating or moderating role of external variables such as policy shifts, market volatility, and technological advances. In addition, while our study emphasizes the contribution of DCs to BMI, the critical role of individual employee contributions (ranging from personal abilities to innovative ideas) in crisis situations deserves further investigation [41]. Future studies should not only explore the personality traits of senior executives but also examine how the capabilities and ideas of employees influence BMI.

Third, while the quantitative methods used in this study revealed certain practices and operational characteristics of SMEs during the pandemic, they lacked the depth provided by qualitative methods, especially in illustrating the causal dynamics between DCs and BMI. Future research could use mixed methods to replicate our findings, thus providing a richer and more detailed understanding of DCs and BMI practices. In addition, given the long-term impact of the pandemic and its varying effects across industries, future research should aim to integrate findings from insights across different industry contexts. This broader perspective could provide valuable experiences and lessons for all market participants, thereby improving their ability to respond to similar crises in the future.

## Supporting information

S1 Data (XLSX)
